# Shear bond strength of adhesive systems on PEEK, PEKK, and fiber-reinforced composites: an *in vitro* study

**DOI:** 10.1186/s12903-026-08096-x

**Published:** 2026-03-17

**Authors:** Esra Bilgi-Ozyetim, Mohammed Sajid Al-Zamily, Sevda Ozturk Yesilirmak

**Affiliations:** 1 Department of Prosthodontics, Faculty of Dentistry, Istanbul Yeni Yuzyil University, Türkiye Sütlüce, Binektaşı Sk. No:10, Istanbul, 34445 Türkiye; 2 Private Clinic, Al Diwaniyah, Iraq; 3 Department of Restorative Dentistry, Faculty of Dentistry, Istanbul Gelişim University, Istanbul, Türkiye

**Keywords:** High-performance polymer, Polyetheretherketone, Polyetherketoneketone, Fiber-reinforced composite, Shear bond strength, Universal adhesives, Adhesion

## Abstract

**Background:**

Strong and durable bonding between composite resins and high-performance polymers (HPPs) is difficult to achieve because of HPPs’ low surface energy and chemical inertness, which limit their clinical applicability. This *in vitro* study aimed to evaluate the shear bond strength (SBS) between a composite resin and HPPs using different adhesive systems.

**Methods:**

Disc-shaped specimens of polyetheretherketone (PEEK), polyetherketoneketone (PEKK), and fiber-reinforced composite (FRC) (*n* = 50 per group) were fabricated and sandblasted with 50 μm aluminum oxide (Al₂O₃) particles. Surface roughness (Ra) was measured using a profilometer, and surface topography was evaluated using scanning electron microscopy (SEM). Each polymer group was divided into five subgroups (*n* = 10) according to the adhesive system used: Scotchbond Universal, G-Premio BOND, Tokuyama Universal Bond, visio.link, and PEKK Bond. Following the adhesive application, a composite resin was applied to each specimen surface and then light-polymerized. All specimens were subjected to thermocycling for 5,000 cycles. After thermocycling, SBS was measured using a universal testing machine. Data were analyzed using a two-way and one-way ANOVA with Tukey’s HSD test (α = 0.05).

**Results:**

FRC demonstrated the highest Ra and SBS values, followed by PEKK and PEEK. The 10-methacryloyloxydecyl-dihydrogenphosphat (MDP) - and silane-containing adhesive (Scotchbond Universal) showed significantly higher SBS values compared to the other adhesive systems. Cohesive failure represented the predominant type of failure in most groups.

**Conclusions:**

Universal adhesives containing functional monomers such as MDP and silane may provide reliable bonding to HPP materials after airborne-particle abrasion. Within the limitations of this *in vitro* study, these findings support the clinical use of universal adhesives for chairside repair and veneering procedures of HPP-based restorations. Additionally, the selection of appropriate surface conditioning and adhesive protocols may help improve the longevity and predictability of polymer-based prosthetic restorations.

## Background

Polyaryletherketone (PAEK) polymers are advanced high-performance polymers (HPPs) composed of aromatic benzene chains linked by functional ether and ketone groups [1–3]. Notably, the ratio of ketone to ether groups in PAEK polymers determines their rigidity, glass transition temperature, and melting point [4]. Although polyetheretherketone (PEEK) is a well-established and widely used PAEK polymer, the recently introduced polyetherketoneketone (PEKK) polymer has exhibited a higher melting point and approximately 80% greater compressive strength. These characteristics are attributed to its higher ketone content and improved solidification of glass and polymer chains [3, 5]. PAEK polymers have also demonstrated favorable stress distribution, good dimensional stability, and high fracture resistance [4, 6], making them promising materials for dental applications.

Zantex (Biofunctional Materials, USA) is a recently developed HPP reinforced with a dense three-dimensional glass fiber network embedded in a polymer matrix [7]. Fiber-reinforced composites (FRCs) offer several advantages, including high biocompatibility, a low elastic modulus, and high fracture strength [8–10]. FRCs used in dentistry typically consist of inorganic particles and fibers that serve as reinforcing compounds, which are bonded to a polymeric matrix by a coupling agent [11–13]. The properties of FRC systems are influenced by several factors, including the type of fiber used, the volume of the fibers in the matrix, the architecture and orientation of the fibers, the type of polymer matrix used, and the interfacial adhesion between the fiber and the matrix [11, 12, 14]. Critically, the manual fabrication of conventional FRCs may lead to voids and weak fiber–matrix bonding, which can compromise their mechanical performance [11, 14, 15]. Therefore, computer-aided design/computer-aided manufacturing (CAD/CAM) FRC systems have gained increased attention for their superior consistency and structural integrity [16].

HPPs are frequently used as framework materials in prosthetic dentistry for removable partial dentures (PEEK), implant-supported fixed prostheses (PEEK, PEKK, FRC), 3-unit fixed partial denture (PEEK, PEKK) endocrowns (PEEK) and resin-bonded fixed partial denture (PEEK) [17]. However, the low translucency and grayish coloration of PAEK polymers [18, 19], as well as the suboptimal optical properties of FRCs, restrict their use in monolithic restorations. This problem is often overcome via veneering frameworks with composite resins using direct or indirect techniques [20, 21]. The inert structure of polymers with low surface energy and resistance to surface modification makes it difficult, however, to reliably adhere veneering materials [22]. In turn, this limitation restricts the widespread use of HPPs in restorative and prosthetic dentistry. Consequently, both chemical and mechanical surface treatment methods have been proposed to enhance the surface properties of HPPs and promote stronger, more durable bonding [23, 24].

Micromechanical and chemical surface modification techniques - including airborne-particle abrasion with aluminum oxide (Al₂O₃) [25], tribochemical silica coating using silica-modified Al₂O₃ [3], etching with sulfuric acid or hydrogen peroxide mixtures [26, 27], plasma treatment [28, 29], and laser surface modification [30] - have been applied to achieve stronger and more durable adhesion to HPPs. Effective bonding is a complex process that depends on different interactions involving chemical, physical, and mechanical factors [2]. Adhesive pretreatment is generally required to chemically condition these inert polymer surfaces before veneering or cementation [31]. Several studies have reported that visio.link material (Bredent, Senden, Germany) provides the highest shear bond strength (SBS) [32–34]. However, this material is only suitable for laboratory use, as it requires a special polymerization furnace for activation, making it unsuitable for chairside applications. To overcome this limitation, researchers have recommended using universal adhesive systems polymerized using a conventional light-emitting diode (LED) to enhance adhesion to PEEK [21, 34, 35].

Despite the growing clinical interest in HPPs, few studies have compared the effectiveness of different adhesives in bonding to these polymers. The bonding performance of Zantex, a recently introduced HPP, to veneering composites has not yet been thoroughly assessed. Previous investigations have mainly focused on methyl methacrylate (MMA)-based adhesive systems, particularly visio.link and PEKK Bond, which have shown promising results in enhancing composite adhesion to PEEK and PEKK. However, the reported outcomes have varied considerably across studies due to differences in polymerization protocols and the frequent use of specialized laboratory equipment. Research on universal adhesives for HPPs is still emerging, and few studies have compared their performance across different polymer types.

To address these gaps, the present study aimed to evaluate and compare the SBS of adhesive materials with different chemical compositions applied to PEEK, PEKK, and FRC under standardized sandblasting conditions. The null hypothesis was that HPPs and different adhesive materials would not significantly affect the SBS of the veneering composite resin.

## Methods

The study was approved by the Istanbul Yeni Yuzyil University Science, Social, and Non-Interventional Health Sciences Research Ethics Committee (approval no: 2023/05-1071). The sample size was determined using a power analysis software (G*Power software, version 3.1.9.4; Heinrich-Heine-University Düsseldorf, Düsseldorf, Germany). Based on an effect size (d) of 0.79 with 80% statistical power (1–β = 0.80) at a 5% significance level (α = 0.05), the minimum required sample size was calculated as 4 specimens per group. To increase the reliability of the results and to account for potential experimental variability, 10 specimens were included in each subgroup.

### Fabrication of specimens

A disc-shaped specimen model (10 mm in diameter and 2 mm in height) was designed using CAD software (EXOCAD, Exocad GmbH, Darmstadt, Germany), converted into a stereolithography (STL) file, and transferred to a CAD/CAM milling unit (Ceramill Motion 2, Amann Girrbach, Germany) for specimen fabrication from prefabricated CAD/CAM HPP discs. In total, 150 specimens were fabricated: 50 from PEEK blanks (BreCAM BioHPP, Bredent GmbH & Co.KG., Senden, Germany), 50 from PEKK blanks (Pekkton Ivory, Cendres Metaux SA, Biel/Bienne, Switzerland), and 50 from FRC blanks (Zantex, Biofunctional Materials, Boca Raton, USA) (Table 1).

**Table 1:** Composition, manufacturer, and lot numbers of the HPP used in the study

	Brand name	Composition	Manufacturer	Lot number
PEEK	BreCAMBioHPP	80% PEEK with 20% nanoceramic filler	Bredent GmbH & Co.KG., Senden, Germany	455939
PEKK	Pekkton Ivory	80% PEKK with 20% filler including titanium dioxide	Cendres Metaux SA, Biel/Bienne, Switzerland	000037702
FRC	Zantex	45% glass-fiber, 28% epoxy resin*	Biofunctional Materials, Boca Raton, USA	E03272

*The remaining composition is not disclosed by the manufacturer

The bonding surface of each specimen was polished using 600- to 1200-grit rotating silicon carbide abrasive papers with a polishing machine (Phoenix Beta Polishing Machine, Buehler, Germany) under running water. All specimens were cleaned for 10 min in an ultrasonic bath containing distilled water and air-dried before surface treatment. Final specimen thickness was controlled using a digital caliper, and all procedures were performed by the same investigator to minimize variability.

All specimens were sandblasted with 50 μm Al₂O₃ particles at 0.5 MPa pressure from a 5 mm distance for 20 s using a sandblasting unit (Renfert Basic, Bremervörde, Germany) in accordance with the method described by Lee et al. [5].

### Surface roughness measurements and SEM evaluations

The surface roughness of each disc-shaped specimen (diameter: 10 mm; thickness: 2 mm) was measured using a contact profilometer (Perthometer M1, Mahr GmbH, Germany). The cut-off length (Lc) was set at 0.25 mm, and the total measuring length (Lt) was set at 1.75 mm. Measurements were performed at 3 separate standardized locations on each specimen surface, and the arithmetic mean surface roughness (Ra) was calculated from these measurements.

One additional specimen from each polymer group (PEEK, PEKK, and FRC) was fabricated for surface morphology analysis. After surface treatment, the specimens were sputter-coated with gold and were analyzed by scanning electron microscopy (SEM) (JEOL JSM-6510 LV SEM, Tokyo, Japan) under ×1.000, x2.000, x3.000, and x5.000 magnifications.

### Bonding procedure

For each material group (PEEK, PEKK, and FRC), the 50 specimens (10 mm in diameter x 2 mm in height) were randomly divided into five experimental subgroups according to the adhesive system used (*n* = 10 per subgroup) (Table 2). All adhesive systems were applied according to the manufacturer’s instructions and were polymerized. Polytetrafluorethylene (PTFE) molds (4 mm inner diameter × 4 mm height) were enterally positioned on each specimen. Similar mold diameters have been reported in previous bonding studies [36–38]. A composite resin (Filtek Z350 XT/ 3 M ESPE Seefeld, Germany), used in a previous bond strength study [5], was placed inside the molds and light-polymerized for 40 s.

**Table 2:** Composition, manufacturer, and lot numbers of the adhesive systems used in the study

Adhesive system	Composition	Manufacturer	Lot number
visio.link (VL)	methyl methacrylate (MMA), 2-propenoic acid reaction products with pentaerythritol (PETIA)	Bredent GmbH, Germany	204809
PEKK Bond (PB)	MMA, Di-urethane dimethacrylate (UDMA), Diphenyl (2,4,6-trimethylbenzoyl) phosphine oxide	Anaxdent GmbH, Germany	2070008
G-premio BOND (GB)	Dimethacrylate (DMA), 10-methacryloyloxydecyl-dihydrogenphosphat (MDP), 4-MET, MDTP	GC Europe, Belgium	2103171
Scotchbond Universal (SB)	MDP, DMA, 2-hydroxyethyl methacrylate (HEMA), Vitrebond™ Copolymer, fillers, ethanol, water, initiators, silane	3 M ESPE, Germany	665259
Tokuyama Universal (TB)	Phosphoric acid monomer, MTU-6, HEMA, bisphenol-A diglycidyl ether dimethacrylate (BisGMA), triethylenglycol dimethacrlyate (TEGDMA), Acetone, y-MPTES, Borate, Peroxide, Acetone, Isopropyl alcohol, Water	Tokuyama dental CO./Japan	802094

### Artificial aging and SBS testing

The specimens were stored in distilled water at 37 °C for 24 h. Thermocycling was performed between 5 °C and 55 °C with a 30-second dwell time for 5,000 cycles in accordance with ISO 10,477 [39].

All specimens were embedded in cold-cured acrylic resin (Imicryl, Imicryl dental, Konya, Turkey), which was poured into cylindrical metal molds to ensure proper fixation in the universal testing machine. An SBS test was performed using a universal testing machine (Instron, Norwood, Massachusetts, USA). After connecting a resin block to the lower holder of the device, the force was applied parallel to the specimen’s bonding surface at a crosshead speed of 1 mm/min until failure occurred. The maximum force value (Newton) was recorded and converted to megapascals (MPa) by dividing the bonding surface area as described in the ISO 10,477 standard (α = P/A) [39].

### Analysis of the failure mode

After the SBS test, the failure modes were examined under a stereomicroscope (Leica MZ7.5, Berlin, Germany) at ×40 magnification. Failure types were classified as adhesive, cohesive, or mixed. Adhesive failure was defined as failure occurring at the interface between the polymer surface and veneering composite resin, cohesive failure was defined as failure occurring within the bulk of the veneering composite resin or adhesive layer, and mixed failure was defined as the simultaneous presence of adhesive and cohesive failure characteristics on the fractured surfaces. The failure mode classification criteria were established according to a previously published bonding study [40].

### Statistical analysis

The statistical analysis was performed using IBM SPSS Statistics version 22. Data normality was assessed using the Shapiro–Wilk and Kolmogorov–Smirnov tests. The combined effects of the polymer type and adhesive system on SBS were analyzed by a two-way ANOVA, followed by Tukey’s HSD post hoc test. A one-way ANOVA was used for comparing surface roughness among polymer groups, and Tukey’s HSD test identified group differences. Failure mode distributions were compared using the chi-square test. The significance level was determined at *p* < 0.05.

## Results

This *in vitro* study evaluated the effects of different HPP materials and adhesive systems on SBS. The results demonstrated statistically significant differences in mean SBS values between the polymer types and adhesive systems (*p*: 0.000; *p* < 0.05). The interaction effect between polymer type and adhesive system on SBS was also statistically significant (*p*: 0.000; *p* < 0.05) (Table 3)^1^.

**Table 3:** Evaluation of the combined effects of high-performance polymer type and adhesive system on shear bond strength

Source	Type III Sum of Squares	df	Mean Square	F	*p*
HPP	204,261	2	102,131	49,241	0,000*
Adesive	366,314	4	91,579	44,153	0,000*
HPPxAdhesive	102,913	8	12,864	6,202	0,000*

### Surface roughness

Surface roughness measurements showed statistically significant differences among the polymer groups (*p*: 0.000; *p* < 0.05). Post hoc analyses revealed that the PEEK group exhibited significantly lower surface roughness than both the PEKK and FRC groups (*p* < 0.05). Additionally, the surface roughness of the PEKK group was significantly lower than that of the FRC group (*p* < 0.05) (Table 4).

**Table 4:** Mean and standard deviation of surface roughness values of high-performance polymers

	Surface Roughness
	Mean ± SD
PEEK	0,69 ± 0,04^A^
PEKK	0,79 ± 0,04^B^
FRC	0,95 ± 0,05^C^
p	0,000*

Oneway ANOVA Test

*p < 0.05

Different capital letters in the columns indicate the difference between polymer groups

### Shear bond strength

When PEEK, PEKK, and FRC polymers were analyzed, statistically significant differences were observed among the adhesive systems in terms of mean SBS values (*p* = 0.000; *p* < 0.05).

In the PEKK and FRC polymer groups, the post hoc analysis revealed that Scotchbond showed significantly higher SBS values than PEKK Bond, G-Premio BOND, Tokuyama Universal Bond, and visio.link (*p* < 0.05). No significant differences were found among the other adhesive systems (*p* > 0.05).

In the PEEK group, the post hoc analysis again demonstrated that Scotchbond exhibited significantly higher SBS values than PEKK Bond, Tokuyama Universal Bond, and visio.link (*p* < 0.05). Moreover, the G-Premio BOND showed significantly higher SBS values compared to PEKK Bond and Tokuyama Universal Bond (*p* < 0.05). No statistically significant differences were observed among the adhesive systems (*p* > 0.05).

When Scotchbond was used, the SBS values of the PEEK group were significantly lower than those of the PEKK and FRC groups (*p* < 0.05). Moreover, the PEKK group showed significantly lower SBS values than the FRC group (*p* < 0.05). Conversely, when PEKK Bond and Tokuyama Universal Bond were used, the SBS values of the PEEK group were significantly lower than those of the PEKK and FRC groups (*p* < 0.05), while no significant difference was observed between the PEKK and FRC groups (*p* > 0.05). Meanwhile, when G-Premio BOND was applied, no statistically significant differences in SBS were observed among the polymer groups. Lastly, for Visio.link, the PEEK group showed significantly lower SBS values than the FRC group (*p* < 0.05) (Table 5).

**Table 5:** Mean and standard deviation of shear bond strength values (MPa) of high-performance polymers according to different adhesive systems

Shear Bond Strength	PEEK	PEKK	FRC
	Mean ± SD	Mean ± SD	Mean ± SD
Scotchbond	12,52 ± 1,49^A, a^	14,74 ± 1,41^B, a^	18,75 ± 2,34^C, a^
PEKK Bond	9,92 ± 1,13^A, b^	11,44 ± 1,47^B, b^	11,83 ± 1,23^B, b^
G-Premio BOND	11,47 ± 0,98^A, ac^	11,81 ± 1,58^A, b^	12,55 ± 2,06^A, b^
Tokuyama Universal Bond	9,84 ± 0,98^A, b^	12,54 ± 1,23^B, b^	13,39 ± 1,42^B, b^
visio.link	10,31 ± 1,37^A, bc^	11,54 ± 1,1^AB, b^	11,81 ± 1,09^B, b^

Two-Way ANOVA Test

Different capital letters in rows indicate the difference between polymer groups

Different lowercase letters in the columns indicate the difference between adhesive groups

### Failure mode

**Fig. 1:**
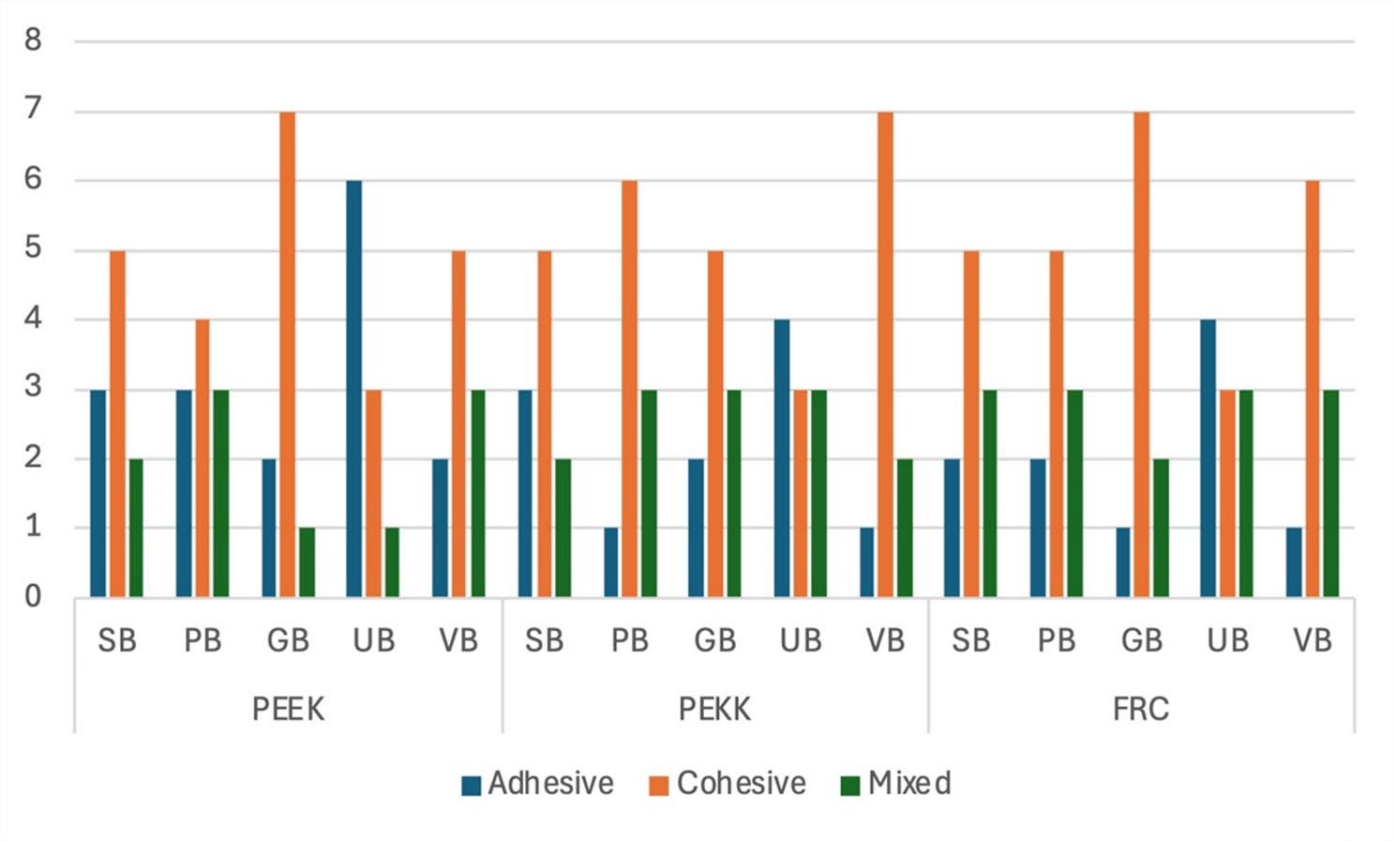
Failure mode distributions of test groups. SB, Scotchbond Universal; PB, PEKK Bond; GB, G-Premio BOND; UB, Tokuyama Universal Bond; VB, visio.link Bond

Within the PEEK, PEKK, and FRC groups, the failure mode analysis revealed no statistically significant differences among the adhesive systems (*p* > 0.05). In all three groups, failure modes were predominantly cohesive, although adhesive and mixed failures were also observed. Notably, a higher rate of adhesive failure was recorded in the Tokuyama Universal Bond subgroup (Fig. 1).

### SEM

**Fig. 2:**
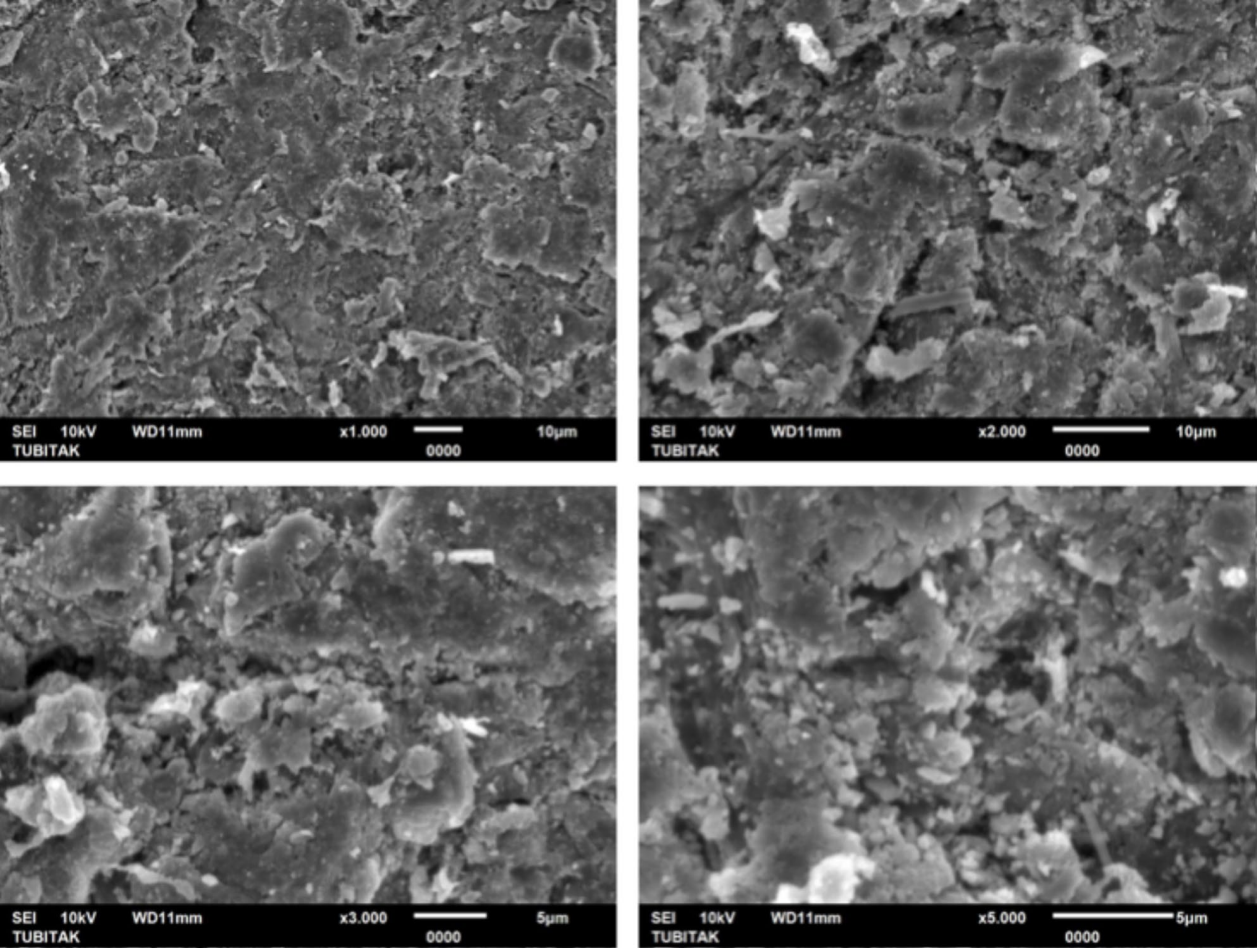
Representative SEM micrographs of sandblasted PEEK surfaces obtained at ×1000, ×2000, ×3000, and ×5000 magnifications

**Fig. 3:**
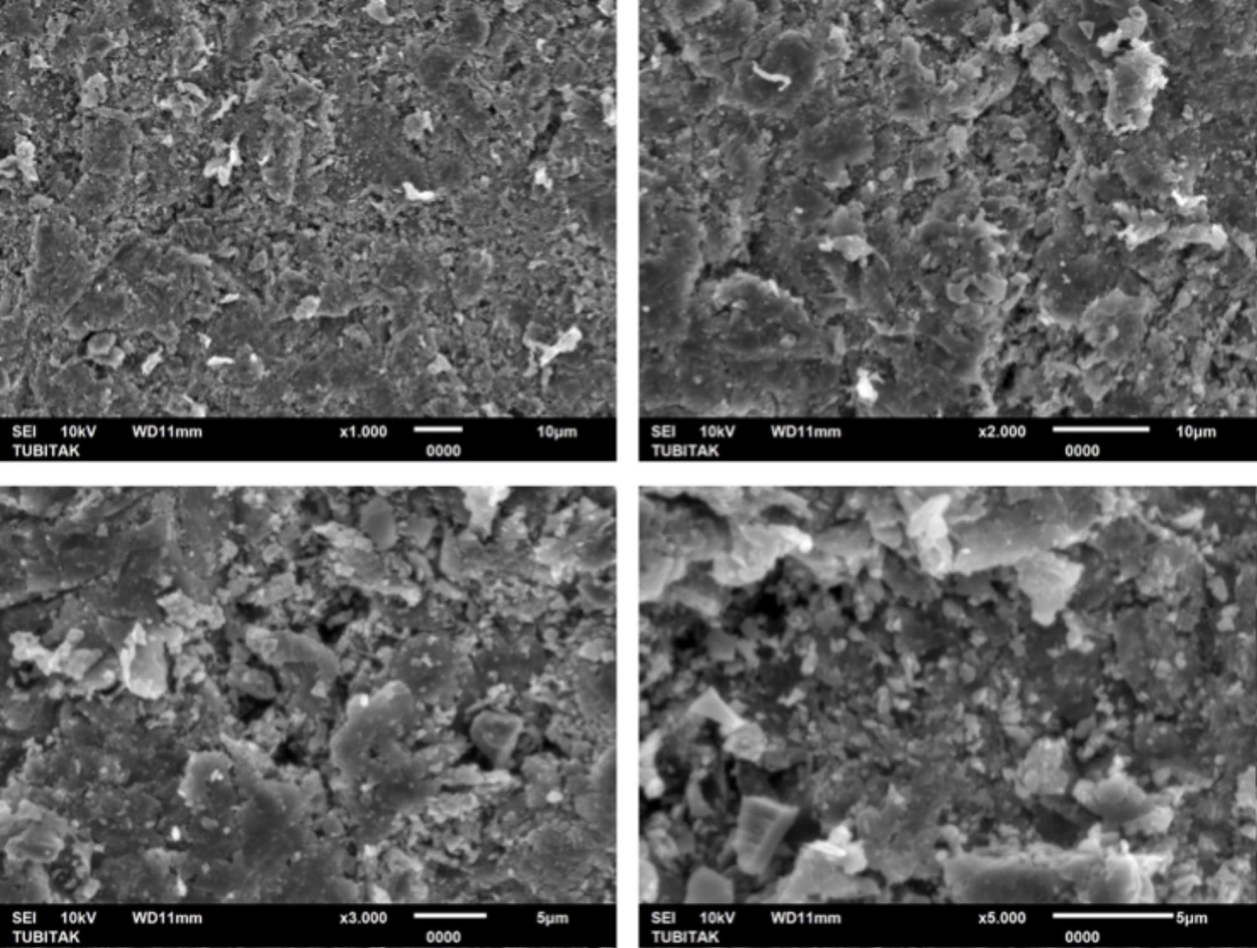
Representative SEM micrographs of sandblasted PEKK surfaces obtained at ×1000, ×2000, ×3000, and ×5000 magnifications

**Fig. 4:**
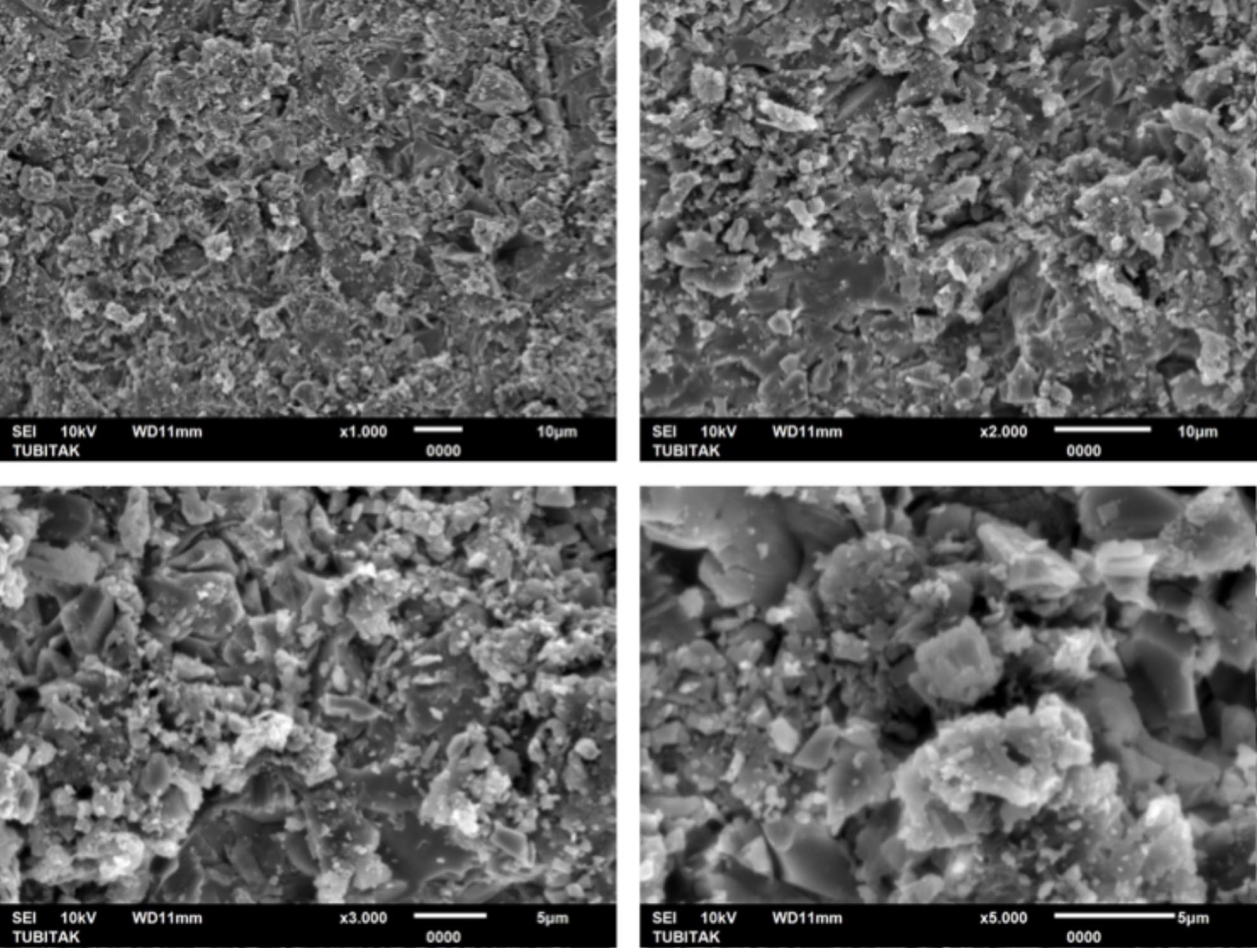
Representative SEM micrographs of sandblasted FRC surfaces obtained at ×1000, ×2000, ×3000, and ×5000 magnifications

At ×3,000 and ×5,000 magnifications, the SEM images showed distinct microtopographic alterations on all polymer surfaces. PEEK exhibited a relatively smooth surface morphology with fine abrasions, whereas PEKK displayed deeper cavities and a rougher texture with sharply defined irregular depressions. FRC revealed the most complex morphology, characterized by rough microstructures and localized recesses within the resin matrix. These observations suggest that the extent and pattern of surface modification were material-dependent, with PEKK and FRC showing more pronounced topographical alterations compared to PEEK (Figs. 2, 3 and 4).

## Discussion

This *in vitro* study evaluated the effect of adhesive systems with different chemical compositions on the bond strength of PEEK, PEKK and FRC polymers to veneering composite resins. The findings revealed statistically significant differences in the mean bond strength values among both the polymer types and the adhesive systems. Therefore, the null hypothesis was rejected.

The surface characteristics of the adherend play a crucial role in adhesive applications, directly affecting bonding performance. Consequently, various surface treatments have been employed to improve the surface properties of polymeric materials and enhance adhesion [25]. Sandblasting, one such approach, is a simple, widely used surface treatment that increases surface roughness, removes organic contaminants, exposes an active surface layer, and enhances the micromechanical interlocking of polymers [6, 41, 42]. Previous studies have reported that sandblasting increases SBS values due to changes in surface morphology [23, 25, 32, 41].

Consistent with previous research, following sandblasting with 50 μm Al₂O₃ particles, this study’s SEM analysis revealed irregular and rough microstructures across all polymer groups, particularly in PEKK and FRC, which correlated with higher bond strength values. Çağlar et al. [37] attributed the high SBS values obtained after sandblasting to modifications in surface topography, where the irregularities in the bonding area increased the effective contact surface available for bonding.

In the present study, both SBS and Ra values were highest in FRC, followed by PEKK, and lowest in PEEK. These results indicated that increased surface roughness enhances micromechanical interlocking and consequently improves adhesive performance. The SEM observations further supported these findings. Notably, PEKK exhibited higher surface roughness and bond strength values than PEEK, consistent with the findings of Lee et al. [5], who reported that air-abrasion more effectively increased surface roughness and wettability in PEKK, leading to superior mechanical retention.

Achieving a reliable bond between the composite resin and the polymer surface remains challenging, representing one of the major clinical limitations of these materials [37, 43]. While previous studies [5, 32, 35, 44, 45] have examined the bond strength between composite resins and HPPs following various surface pretreatments and adhesive systems with different chemical compositions, the reported findings remain inconsistent and sometimes contradictory. Moreover, to the knowledge of the authors, limited evidence exists regarding the bonding behavior of newly developed FRC polymers such as Zantex.

In the present study, the SBS values of the Scotchbond Universal adhesive applied to PEEK, PEKK, and FRC were recorded as 12.52 MPa, 14.74 MPa, and 18.75 MPa, respectively. Scotchbond Universal exhibited the highest SBS on FRC surfaces, followed by PEKK, while the lowest value was observed on PEEK.

Previous studies have shown variable outcomes regarding the bonding performance of Scotchbond Universal to HPPs. Tsuka et al. [44] found a lower SBS value (6.8 MPa) for Scotchbond Universal on PEEK after 24 h of water storage. On the other hand, Bunz et al. [35] reported that after 5,000 thermocycling cycles, Scotchbond Universal produced higher SBS values on both PEEK (13.85 MPa) and FRC (11.49 MPa) surfaces than other adhesive systems, suggesting its effectiveness as a bonding agent for both polymers. While the SBS value for PEEK reported by Bunz et al. [35] was comparable with the present findings, Bunz et al. [35] reported markedly lower SBS values for FRC than those observed in the present study. This discrepancy is likely related to differences in the chemical composition of the FRC materials used in the two studies. The Zantex material used in the current research contained approximately 28 wt% crystalline inorganic compounds and 45 wt% continuous glass fibers arranged in a multilayered, bidirectional configuration within an epoxy resin matrix [9]. In composite veneers bonded to FRC, the bonding interface is formed by the exposed glass fibers and the polymer matrix; therefore, surface pretreatment plays a crucial role in enhancing bond strength at the FRC–composite interface [46].

Lee et al. [5], meanwhile, investigated PEKK surfaces and reported that an adhesive agent containing both 10-methacryloyloxydecyl-dihydrogenphosphat (MDP) and silane (Single Bond Universal) achieved high shear bond strength regardless of surface treatment. They further observed that MDP-containing adhesives, such as All-Bond Universal (BISCO) and Single Bond Universal (3 M ESPE), showed no statistically significant differences in SBS compared with the MMA-based adhesive, which they attributed to the fact that MDP has a similar effect to MMA-containing bond materials on a roughened PEKK surface. This similar effect likely stems from MDP’s durability; owing to its hydrophobic methacrylate and hydrophilic phosphate terminal groups, MDP functions as a bifunctional adhesive monomer capable of forming chemical bonds with both copolymerizable resin monomers and oxide layers, thereby promoting durable adhesion [47–49]. This mechanism may also help explain the relatively high bond strength values obtained with MDP-containing adhesives in the present study.

Whereas both Scotchbond Universal and G-Premio BOND used in this study contain MDP; however, Scotchbond Universal has an additional silane component in its formulation, which may explain its higher SBS values compared with G-Premio BOND. Consistent with previous findings [5, 7, 50], the present study also confirmed that silane contributes significantly to the bonding performance of HPPs when incorporated as a functional component of adhesive systems.

In this study, Tokuyama Universal Bond achieved SBS values of 13.39 MPa for FRC, 12.54 MPa for PEKK, and 9.84 MPa for PEEK. This outcome may stem from its formulation containing phosphate-functional 3D-SR monomers and γ-MPTES silane coupling agents, which are designed to enhance chemical interaction and improve bonding stability according to the manufacturer’s specifications [51]. The relatively uniform performance of Universal Bond across different HPP substrates observed in this study indicates that its formulation can provide consistent adhesion, even on surfaces with limited oxide content; however, further research is needed to confirm these findings and clarify the underlying bonding mechanisms.

Previous studies [21, 37, 45] have shown that adhesive systems containing MMA monomers improve the bonding performance between PEEK and resin composites. For instance, Çağlar et al. [37] obtained the highest SBS values ​​(19.86 ± 2.52 MPa) in the group treated with sandblasting and visio.link. Kern and Lehmann [45] likewise achieved the highest bond strength values ​with adhesive materials containing MMA. Similarly, Stawarczyk et al. [21] confirmed that an MMA-containing adhesive exhibited the greatest bonding performance to PEEK. In contrast, in the present study, visio.link showed lower SBS values than Scotchbond Universal across the PEEK (10.31 MPa), PEKK (11.54 MPa), and FRC (11.81 MPa) groups. However, no statistically significant difference was found among the other adhesive systems. Similarly, PEKKBond exhibited lower SBS values than Scotchbond Universal in the PEKK (11.44 MPa) and FRC (11.83 MPa) groups and lower values than both Scotchbond Universal and G-Premio BOND in the PEEK group (9.92 MPa). Nevertheless, these differences were not statistically significant among the remaining adhesives. Although the manufacturers recommend different polymerization protocols, previous reports have used both laboratory polymerization units and conventional LED curing devices for visio.link and PEKK Bond. In this study, all adhesives were polymerized under standardized LED curing conditions to ensure methodological consistency and to better reflect chairside applicability. The variation in SBS results across studies may be explained by several methodological and material-related factors. In particular, differences in polymerization protocols, surface-treatment parameters, and the intrinsic chemical or structural characteristics of HPP substrates can all influence bonding performance.

In accordance with ISO 10,477, an SBS value of at least 5 MPa between resin-based materials and their respective substrates is regarded as the minimum acceptable threshold for adhesion [39]. Furthermore, minimum clinical thresholds of approximately 10–12 MPa have been proposed for intraoral conditions [52]. In the present study, all groups satisfied this ISO 10,477 criterion. Additionally, except for the PEEK groups treated with PEKK Bond and Tokuyama Universal Bond, the clinically recommended SBS threshold was also achieved. The findings of this study indicate that, following 50 μm Al₂O₃ sandblasting, universal adhesive systems polymerized with conventional LED curing units can achieve clinically acceptable and durable bond strength. This is particularly relevant for chairside repair of HPP-based prostheses.

In this study, no statistically significant differences were observed among the adhesive systems in terms of failure types across the PEEK, PEKK, and FRC groups. However, specimens treated with Tokuyama Universal Bond predominantly exhibited adhesive failures, whereas the other adhesive groups more frequently exhibited cohesive failures. This finding suggests that despite the relatively higher bond strength achieved with Universal Bond, particularly on PEKK and FRC surfaces, the interfacial adhesion remained weaker than the cohesive strength of the substrate or composite, resulting in interfacial separation during debonding.

This study has several limitations. As an *in vitro* study, the findings may not fully reflect the complex biomechanical and environmental conditions of the oral cavity. In addition, although thermal cycling was performed to simulate intraoral temperature changes, mechanical aging procedures, which may influence long-term bonding behavior, were not included. SBS was evaluated only after thermocycling, but evaluating bond strength both before and after aging procedures may provide better insight into the bonding behavior of polymer-based materials. Furthermore, the absence of an untreated control group may limit the evaluation of baseline bonding performance and the overall effect of surface treatment and adhesive protocols. In addition, the SEM evaluation was limited to surface-treated specimens, and untreated polymer surface morphology was not examined. The evaluation of both treated and untreated polymer surfaces under SEM may help demonstrate the effect of surface treatment on surface topography and bonding performance.

Lastly, the limited number of studies comparing the effects of universal adhesive systems with different chemical compositions on HPP surfaces, as well as the limited number of studies evaluating the bond strength of FRC materials, has made it difficult to directly compare and interpret the present findings within the existing literature. While some findings could be interpreted in relation to prior research, this gap in the literature indicates a clear need for further research.

To address some of the limitations of this study future investigations should focus on evaluating bonding performance under combined thermal and mechanical aging conditions to better simulate intraoral functional stresses. In addition, assessing SBS both before and after aging procedures would provide a more comprehensive understanding of the bonding mechanisms and durability of adhesive interfaces in polymer-based restorative materials. Such data would contribute to improving the predictability of repair protocols and the long-term clinical performance of HPP restorations.

## Conclusion

Within the limitations of this *in vitro* study, both the adhesive system and the type of HPP significantly influenced bonding performance to veneering composite resins. Among the evaluated materials, the highest bond strength and surface roughness values were observed in the FRC group. Scotchbond Universal, which contains both MDP and silane functional components, demonstrated the highest bonding performance. These findings suggest that universal adhesive systems, when combined with appropriate surface pretreatment, may provide clinically acceptable bonding to high-performance polymers. From a practical perspective, these findings may support the clinical use of universal adhesives as an alternative to laboratory-based bonding systems, particularly for chairside repair and veneering procedures of HPP-based restorations.

## Data Availability

The datasets used and/or analyzed during the current study are available from the corresponding author on reasonable request.

## Footnotes

1 *Two-Way ANOVA Test                            *p<0.05*

## Abbreviations

Al₂O₃: Aluminum oxide
BisGMA: Bisphenol-A diglycidyl ether dimethacrylate
CAD/CAM: Computer-aided design/computer-aided manufacturing
DMA: Dimethacrylate
FRC: Fiber-reinforced composite
HEMA: 2-hydroxyethyl methacrylate
HPP: High-performance polymers
Lc: Cut-off length
LED: Light-emitting diode
Lt: Measuring length
MDP: 10-methacryloyloxydecyl-dihydrogenphosphat
MMA: Methyl methacrylate
PEEK: Polyetheretherketone
PEKK: Polyetherketoneketone
PETIA: 2-propenoic acid reaction products with pentaerythritol
PTFE: Polytetrafluorethylene
Ra: Surface roughness
SBS: Shear bond strength
SEM: Scanning electron microscopy
TEGDMA: Triethylenglycol dimethacrlyate
UDMA: Di-urethane dimethacrylate
